# Microglial FoxO3a deficiency ameliorates ferroptosis‐induced brain injury of intracerebral haemorrhage via regulating autophagy and heme oxygenase‐1

**DOI:** 10.1111/jcmm.18007

**Published:** 2023-10-27

**Authors:** Rikang Wang, Zhi Liang, Xiaoyan Xue, Hua Mei, Lianru Ji, Bocheng Wang, Wenjin Chen, Chao Gao, Shun Yuan, Tao Wu, Hui Qi, Suifa Hu, Li Yi, Yonggui Song, Rifang Liao, Baodong Chen

**Affiliations:** ^1^ Department of Neurosurgery Peking University Shenzhen Hospital Shenzhen China; ^2^ Jiangxi University of Chinese Medicine Nanchang China; ^3^ Ganzhou People's Hospital Ganzhou China; ^4^ Department of Pharmacy Guangdong No.2 Provincial People's Hospital Guangzhou China; ^5^ Department of Pharmacy, Sun Yat‐sen Memorial Hospital Sun Yat‐sen University Guangzhou China

**Keywords:** ferroptosis, FoxO3a, HO‐1, intracerebral haemorrhage, microglia

## Abstract

Microglial HO‐1 regulates iron metabolism in the brain. Intracerebral haemorrhage (ICH) shares features of ferroptosis and necroptosis; hemin is an oxidized product of haemoglobin from lysed red blood cells, leading to secondary injury. However, little is known about the underlying molecular mechanisms attributable to secondary injury by hemin or ICH. In this study, we first show that FoxO3a was highly co‐located with neurons and microglia but not astrocytes area of ICH model mice. Hemin activated FoxO3a/ATG‐mediated autophagy and HO‐1 signalling resulting in ferroptosis in vitro and in a mice model of brain haemorrhage. Accordingly, autophagy inhibitor Baf‐A1 or HO‐1 inhibitor ZnPP protected against hemin‐induced ferroptosis. Hemin promoted ferroptosis of neuronal cells via FoxO3a/ATG‐mediated autophagy and HO‐1 signalling pathway. Knock‐down of FoxO3a inhibited autophagy and prevented hemin‐induced ferroptosis dependent of HO‐1 signalling. We first showed that hemin stimulated microglial FoxO3a/HO‐1 expression and enhanced the microglial polarisation towards the M1 phenotype, while knockdown of microglial FoxO3a inhibited pro‐inflammatory cytokine production in microglia. Furthermore, the microglia activation in the striatum showed significant along with a high expression level of FoxO3a in the ICH mice. We found that conditional knockout of FoxO3a in microglia in mice alleviated neurological deficits and microglia activation as well as ferroptosis‐induced striatum injury in the autologous blood‐induced ICH model. We demonstrate, for the first time, that FoxO3a/ATG‐mediated autophagy and HO‐1 play an important role in microglial activation and ferroptosis‐induced striatum injury of ICH, identifying a new therapeutic avenue for the treatment of ICH.

## 1 INTRODUCTION

Intracerebral haemorrhage (ICH) is a common acute clinical cerebrovascular disease. In many studies, the 30‐day mortality rate of ICH was 32%–52%, Half of the deaths occurred within the first 2 days.^1^ Some evidence suggests that the mortality rate has declined, which may be due to better supportive treatment and secondary prevention.^2^ However, the decline in mortality may be due to an increase in the proportion of survivors with residual disabilities to a certain extent.^1^, ^3^ Therefore, we try to find new pathological mechanisms of ICH and new therapeutic targets.

Neuro‐inflammation plays an important role in the pathogenesis of ICH. The innate immune cell of the brain is considered to be the first batch of cells to respond to various acute brain injuries, including ICH.^4^, ^5^ After ICH, overproduction of iron associated with the induction of heme oxygenase‐1 (HO‐1) in the brain was observed.^6^, ^7^, ^8^ However, the role of HO‐1 after ICH is controversial. Some studies showed that induction of HO‐1 could provide beneficial effects due to its anti‐inflammatory and antioxidant.^9^, ^10^ Overexpression of HO‐1 in the astrocyte improves outcome after ICH.^11^ Whereas others have shown that inhibiting HO‐1 reduces brain injury and improves ICH outcomes.^10^, ^12^, ^13^ In addition, prolonged induction of HO‐1 was significantly increasing the recovery time of ICH mice. Nevertheless, increased expression of HO‐1 was associated with a deleterious iron accumulation in activated microglia in a rodent stroke model.^14^ A recent study showed that HO‐1 deletion reduces iron deposition/toxicity in a mouse model of ICH,^15^ HO‐1 overexpression contributes to neurotoxic iron accumulation providing deleterious effects in aged mice exposed to an inflammatory insult.^16^ The functional regulation between FoxO3a and HO‐1 in microglia activation and ferroptosis‐induced brain injury of ICH has not been elucidated yet. Heme oxygenase‐1 had binding sites to FoxO3a based on bioinformatics analysis and was abnormally expressed and positively related to FoxO3a.^17^ Given the aforementioned association, we speculated that FoxO3a may regulate neuronal ferroptosis in ICH via binding to the HO‐1 promoters and believe the verification of this hypothesis is conducive to ICH treatment.

Notably, FoxO3a promotes traumatic brain damage by regulating neuronal autophagy and oxidative stress and is implicated in nerve damage and inflammatory responses in ICH.^18^, ^19^ A recent study found inhibition of FoxO3a/ATG‐mediated autophagy ameliorates hippocampal injury after ICH^19^. Another study found that suppression of autophagy effectively attenuated post‐stroke hippocampal inflammation and neuronal damage.^20^ Although few studies have shown that silencing FoxO3a attenuates ICH‐induced neuronal ferroptosis and ameliorates post‐ICH brain damage,^18^ however, whether microglial FoxO3a/ATG‐mediated autophagy is involved in neuro‐inflammation and ferroptosis‐induced striatum injury of ICH remains largely unknown.

In this study, we investigated the role of FoxO3‐mediated autophagy and HO‐1 in the inflammatory process and ferroptosis‐induced striatum injury of ICH. The results of the current study might shed light on the enigma of inflammation and facilitate the improvement of therapeutic strategies for ICH patients.

## 2 MATERIALS AND METHODS

### 2.1 Cell culture and reagents

BV‐2 and HT22 were obtained from the China Center for Type Culture Collection and cultured in DMEM (11995; Solarbio Co., Ltd.) containing 10% foetal bovine serum (40130ES76; Yeasen Co., Ltd.), supplemented in acceptable condition at 37°C, 5% CO_2_. Cells were seeded at 5 × 10^4^ cells/mL. When the cells were confluent to 70%, hemin (H9039; Sigma‐Aldrich Co., Ltd.), Baf‐A1 (HY‐100558; MedChemExpress Co., Ltd.) and ZnPP (HY‐101193; MedChemExpress Co., Ltd.) were used in this experiment.

### 2.2 Animals and genotyping

Both FoxO3a CKO^CX3CR1^ mice and C57BL/6J (Wild‐type) mice were used as experimental animals. We used the FoxO3a^fl/fl^ mice (Cyagen Biosciences Co., Ltd.) and the CX3CR1^creER^ mice (Biocytogen Pharmaceuticals Co., Ltd.) for breeding. Utilising a MiniBEST Universal Genomic DNA Extraction Kit, genomic DNA was extracted from tail tips (9765; Takara Bio Inc.). According to the manufacturer's instructions, the PCR reaction was carried out using Taq Master Mix (P213‐02; Vazyme Biotech Co., Ltd.) and the specific primers. Tamoxifen (75 mg/kg) (HY‐13757A; Med Chem Express Co., Ltd.) was injected intraperitoneally into adult mice for 5 days in a row. One week after tamoxifen administration, all related animal experiments were conducted. Experimental animals were maintained in a specific pathogen‐free environment of controlled temperature (21–24°C) and 12‐h light/12‐h dark cycle. All animal procedures were conducted according to the Jiangxi University of Chinese Medicine Laboratory Animal Science and Technology Center's institutional animal care and use committee.

### 2.3 Lipid peroxidation detection

HT22 cells were seeded in 6‐well plates. After stimulation with different concentrations of hemin, cells were stained with 2 μM C11‐BODIPY (581/591) probe (D3861; Invitrogen Co., Ltd.) in accordance with the manufacturer's instructions. Cells were visualised under the fluorescence microscope (DM IL, Leica Co., Ltd.). Oxidised and reduced types were observed at excitation/emission wavelengths of 488/510 nm (FITC filter set) and 581/591 nm (TRITC filter set).

### 2.4 Western blots analysis

Cells were lysed in ice‐cold RIPA buffer containing protease inhibitor. The same amounts of protein were separated using an SDS‐PAGE gel and transferred to PVDF membranes (R1KB43643; Merck Co., Ltd.). The membranes were blocked in 5% skim milk (D8340; Solarbio Co., Ltd.) and then incubated overnight at 4°C with the primary antibodies for FoxO3a (66428‐1‐Ig; Proteintech Co., Ltd.), FoxO1 (18592‐1‐AP; Proteintech Co., Ltd.). Arg‐1 (66129‐1‐Ig; Proteintech Co., Ltd.), CD206 (60143‐1‐Ig; Proteintech Co., Ltd.), CD86 (13395‐1‐AP; Proteintech Co., Ltd.), GPX4 (A11243; ABclonnal Co., Ltd.), FTH1 (A19544; ABclonnal Co., Ltd.), HO‐1 (A19062; ABclonnal Co., Ltd.), LC3 (14600‐1‐AP; Proteintech Co., Ltd.), NCOA4 (A17330; ABclonnal Co., Ltd.), SLC7A11 (A2413; ABclonnal Co., Ltd.), β‐actin (HC201‐01; Transgen Co., Ltd.). Then subjected to HRP‐labelled secondary antibody for 1 h at room temperature. The reaction was visualised with chemiluminescent assay.

### 2.5 Cell viability assay

Cell viability was determined by the CCK‐8 assay kit (MA0218; Meilunbio Co., Ltd.). Following the manufacturer's instructions, the drug‐treated cell supernatant was replaced with complete medium containing 10% CCK‐8 enhancer, and after incubation at 37°C for 2 h, the absorbance was measured with a microplate reader at a wavelength of 405 nm. Three replicates were performed within each group, and the average value of the control group was used as the baseline to count cell viability.

### 2.6 Intracranial injection of adeno‐associated virus

To knock down FoxO3a, pAAV‐U6‐shRNA (FoxO3a)‐CMV‐EGFP‐WPRE and pAAV‐U6‐shRNA (NC2)‐CMV‐EGFP‐WPRE viruses (Obio Technology Co., Ltd.) were used in this experiment. AAV injection using 6‐week‐old C57BL/6 mice, 1 μL of adeno‐associated virus was injected into the mouse striatum at 0.2 μL/min using a stereotaxic apparatus (RWD Co., Ltd.; AP = −0.2, ML = +2.3, DV = −3.6). Intracerebral haemorrhage modelling experiments were performed 3 weeks after adeno‐associated virus expression.

### 2.7 Mouse model of ICH

Mice were anaesthetised by intraperitoneal injection of sodium pentobarbital, then fixed in a stereotactic instrument and injected the autologous blood into mouse striatum (AP = −0.2, ML = +2.3, DV = −3.6) at a rate of 1 μL/min. The mouse in the sham group was injected with the same volume of saline. The mice were kept warm with an electric heating pad after surgery.

### 2.8 Behavioural analysis

#### 2.8.1 Forelimb placing test

Gently handle the mouse until it relaxes to examiner's touch. The palm should be lightly in contact with the dorsal side of the animal's torso, and the thumb and fingers should firmly grasp the sides of the head. The forelimb not being examined should be securely and safely trapped underneath by the examiner's middle and ring fingers around the animal's anterior ribcage. The animal's rear limbs can be supported, and its body can be held in place while testing using the opposite hand. Record the number of times the mouse puts the forelimb on the table and calculate the percentage.

#### 2.8.2 Corner turn test

The corner turn apparatus consists of two walls forming a 30° corner. The examiner let the mice enter the test area naturally, and recorded the direction of left and right turns of the mice, and carried out a total of ten tests to calculate the percentage of turns.

### 2.9 Small interfering RNA transfection

The small interfering (si) RNA oligonucleotides for FoxO3a were designed and acquired from GenPharma. To silence gene expression, cell transfection was performed with Lipofectamine 3000 reagent (L3000015; Thermo Fisher Scientific Co., Ltd.) according to the manufacturer's directions. The sequences of FoxO3a were shown as follows: sense: 5′‐GGAACGUGAUGCUUCGCAATT‐3′, antisense: 5′‐UUGCGAAGCAUCACGUUCCTT‐3′.

### 2.10 Immunofluorescence staining

The brain tissues were fixed with 4% polyformaldehyde for 24 h. And then they were collected in 30% sucrose solution (S112228; Rhawn Co., Ltd.) for 48 h before being snapped free and cryosectioned at 20 μm thickness. Sections were incubated in 0.5% Triton X‐100 (T8200; Solarbio Co., Ltd.) for 20 min and blocked with PBS containing 3% goat serum (SL038; Solarbio Co., Ltd.) for 1 h at room temperature before permeating overnight at 4°C with primary antibodies. The brain tissues were then incubated with primary antibodies against IBA1 (019‐19741; Wako Pure Chemical Industries, Co., Ltd.), FoxO3a (66428‐1‐Ig; Proteintech Co., Ltd.), GPX4 (A11243; ABclonnal Co., Ltd.), LC3 (14600‐1‐AP; Proteintech Co., Ltd.), NeuN (26975‐1‐AP; Proteintech Co., Ltd.), 4‐HNE (bs‐6313R; Bioss Co., Ltd.) overnight at 4°C. After that, DyLight 549‐labelled goal anti‐mouse IgG (SA00013‐7; Proteintech Co., Ltd.) and DyLight 488‐labelled goal anti‐rabbit IgG (SA00003‐4; Proteintech Co., Ltd.) were added and incubated at 37°C for 1 h, and the nucleus was stained by DAPI (S2110; Solarbio Co., Ltd.). The cells were imaged using an inverted fluorescence microscope (DM IL, Leica Co., Ltd.).

### 2.11 RT‐PCR analysis

The cells and brain tissue RNA was synthesised using TRIzon reagent (CW0580; CWBIO Co., Ltd.), and 0.2ug of total RNA was used to synthesize. The first strand of cDNA is synthesised using the 1st Strand cDNA Synthesis reagent (11141ES60; Yeasen Biotech Co., Ltd.). Then, the cDNA was amplified with SYBR Green PCR Master Mix (11201ES08; Yeasen Biotech Co., Ltd.) in a Roche LightCycler®96 System (Roche Co., Ltd.). The relative standard curve was calculated using $2^{-∆∆\mathrm{CT}}$ method, and GAPDH was used as a housekeeping gene for internal normalisation. All primers were following the Table 1.

### 2.12 Perl's iron stain

Using the Prussian Blue Iron Stain Kit (G1422; Solarbio Co., Ltd.) according to the reagent supplier's instructions. Paraffin sections of 5 μm mouse brain tissue was routinely dewaxed and hydrated, then stained using Perl's Blue A and B solutions followed by xylene clear and neutral resin sealing. Then, sections were examined and photographed under transmitted light microscopy in a Leica microscope (DM3000; Leica Co., Ltd.) using a 40× objective.

### 2.13 Statistical analysis

GraphPad Prism software (Version 9.0) was used to perform statistical tests. Results are displayed as means ± the standard deviation (SD) for continuous data and as frequencies for categorical data. The statistical significance of the differences between groups was determined using the T‐test and one‐way anova. *p* values < 0.05 were taken to indicate significant differences.

## 3 RESULTS

### 3.1 The expression of FoxO3a were increased in neuronal cells and in the ipsilateral striatum after ICH

**FIGURE 1:**
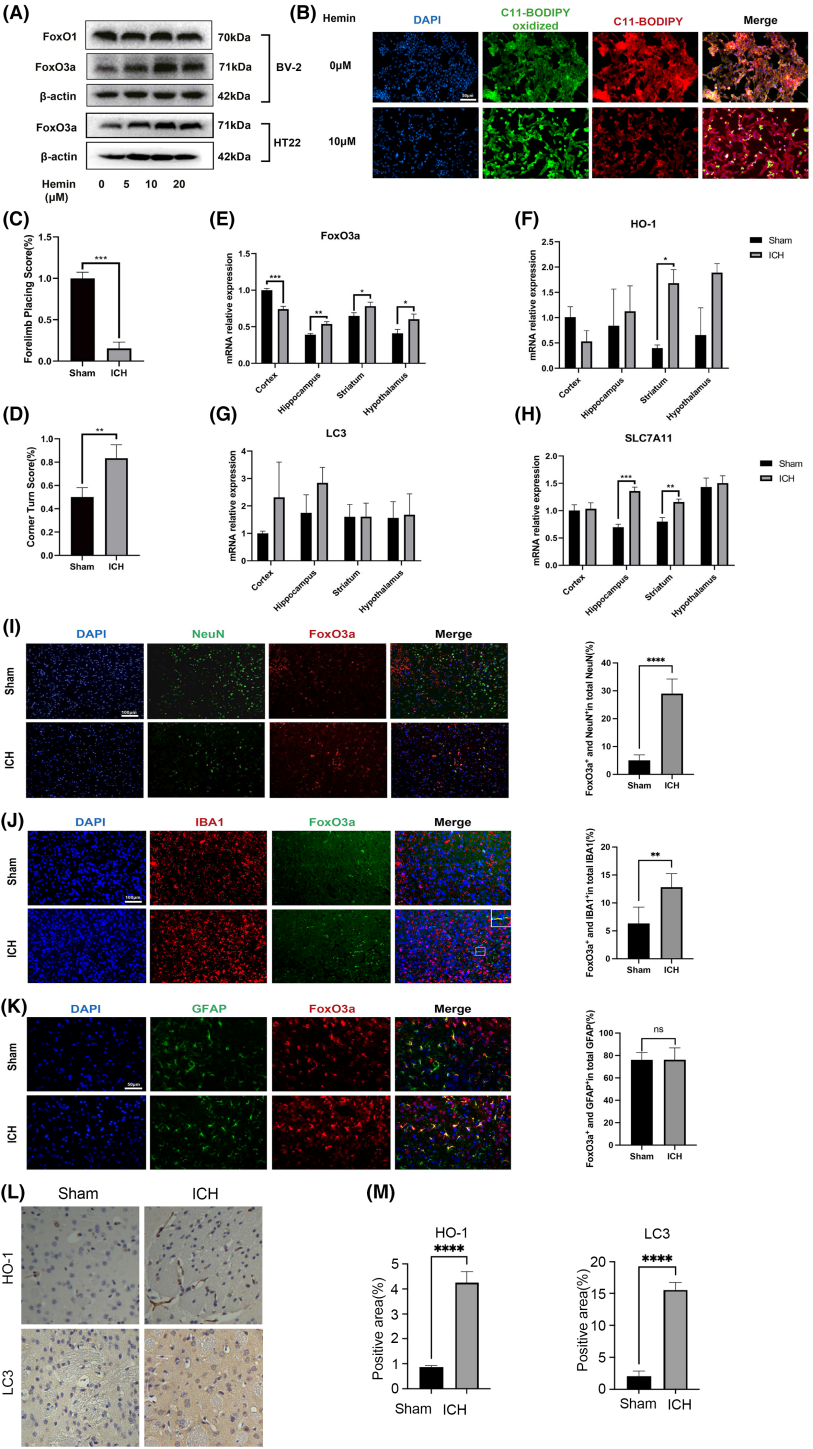
The expression of FoxO3a is increased after treatment with hemin in vitro and in an ICH mice model. (A) HT22 cells and BV‐2 cells were treated with different concentrations of hemin, and the expressions of FoxO1/FoxO3a were determined by western blot (B) The lipid peroxidation was detected by C11 BODIPY 581/591 probe in HT22 cells after stimulated with 20 μM hemin for 24 h. (C) The forelimb placing test and (D) corner turn test of the mice in ICH model by injection of autologous whole blood (*n* = 5). (D) Representative immunofluorescence images of FoxO3a expression in the striatal region caused by ICH. (E–H) The mRNA levels of FoxO3a/HO‐1/LC3‐II/SLC7A11 in the cortex, hippocampus, striatum and hypothalamus of the mice in ICH model. (I–K) Representative images of FoxO3a expression in the striatal area visualised under microscopy. Quantification of co‐localization of NeuN, GFAP or Iba1 with FoxO3a in the striatum of mice under normal conditions or after ICH (*n* = 5). (L) Representative images of IHC staining for HO‐1/LC3 in the striatum of mice under normal conditions or after ICH (*n* = 3). (M) Graphical results showing the IHC scores for HO‐1/LC3 positive area. Data were expressed as mean ± SD. *p < 0.05, **p < 0.01, ***p < 0.001, ****p < 0.0001.

To determine the role of FoxO3a in nerve cell ferroptosis in vitro, HT22 cells and BV‐2 cells were treated with hemin to establish the in vitro ICH model. When different concentration of hemin (5, 10 and 20 μM) were used to treat HT22 cells and BV‐2 cells for 24 h, we found that hemin stimulated FoxO3a protein levels in concentration‐dependent manner, whereas hemin had no effect on FoxO1 expression in BV‐2 cells (Figure 1A). We used C11‐BODIY581/591 probes to detect lipid peroxidation; the green fluorescence indicated oxidation type, and red fluorescence indicated non‐oxidation type. We found hemin‐induced lipid peroxidation in HT22 cells (Figure1B).

**TABLE 1:** All primers in RT‐PCR experiment as following.

Gene	Direction	Primer sequence, 5′‐3′
GAPDH	Sense	AAGGTCGGTGTGAACGGATT
	Antisense	TGAGTGGAGTCATACTGGAACAT
SLC7A11	Sense	AGGGCATACTCCAGAACACG
	Antisense	GGACCAAAGACCTCCAGAATG
GPX4	Sense	TAAGAACGGCTGCGTGGTGAAG
	Antisense	AGAGATAGCACGGCAGGTCCTT
TFR1	Sense	GTTTCTGCCAGCCCCTTATTAT
	Antisense	GCAAGGAAAGGATATGCAGCA
LC3‐II	Sense	AGCTCTGAAGGCAACAGCAACA
	Antisense	GCTCCATGCAGGTAGCAGGAA
FoxO3a	Sense	CTCTCAGGCTCCTCACTGTA
	Antisense	ATGAGTTCACTACGGATGAT
IL‐1β	Sense	CTGCAAGAGACTTCCATCCAG
	Antisense	ATACTGCCTGCCTGAAGCTCTTGT
IL‐6	Sense	CTGCAAGAGACTTCCATCCAG
	Antisense	AGTGGTATAGACAGGTCTGTTGG
CD86	Sense	TCAGTATCTCCAACAGCCTCTC
	Antisense	TCCAGAACACACACAACGGT
CD206	Sense	CAAGGAAGGTTGGCATTTGT
	Antisense	CCTTTCAGTCCTTTGCAAGC
Arg‐1	Sense	AAGAATGGAAGAGTCAGTGTGG
	Antisense	GGGAGTGTTGATGTCAGTGTG
HO‐1	Sense	AATGTGGCCTTCTCTCTGTAAGGG
	Antisense	TGGTTTCAAAGTTCAGGCCACTGG
ATG5	Sense	TGTGCTTCGAGATGTGTGGTT
	Antisense	ACCAACGTCAAATAGCTGACTC
ATG7	Sense	TCTGGGAAGCCATAAAGTCAGG
	Antisense	GCGAAGGTCAGGAGCAGAA
ATG12	Sense	TGAATCAGTCCTTTGCCCCT
	Antisense	CATGCCTGGGATTTGCAGT

To make an ICH model, the mice were injected autologous blood into the striatum. In comparison with the control group (the sham operation group), the ICH model mice have severe neurological dysfunction and weakened motor coordination (Figure 1C,D). We performed real‐time PCR experiments on the cortex, hippocampus, striatum and hypothalamus of the mice in ICH model. It was found that the expression of FoxO3a was significantly increased in the hippocampus, striatum and hypothalamus after ICH, but the expression in the cortex was relatively decreased. In ICH model mice, the autophagy gene LC3 tended to be elevated in the cortex and hippocampus, whereas the expression of HO‐1 was significantly elevated only in the striatum. The expression of the ferroptosis‐related gene SLC7A11 was increased in the hippocampus and striatum (Figure 1E–H). FoxO3a was highly co‐localised with NeuN+ neurons and Iba‐1+ microglia, but no significant GFAP‐positive astrocyte area in ICH model mice (Figure 1I–K). This data suggests that high FoxO3a expression in neurons and microglia may be related to ICH. Immunohistochemical (IHC) staining confirmed the result that HO‐1 and LC3 expressions were increased in the striatum after ICH (Figure 1L,M).

### 3.2 FoxO3a promoted neuronal and microglial ferroptosis‐induced by hemin through HO‐1 and the autophagy pathway

**FIGURE 2:**
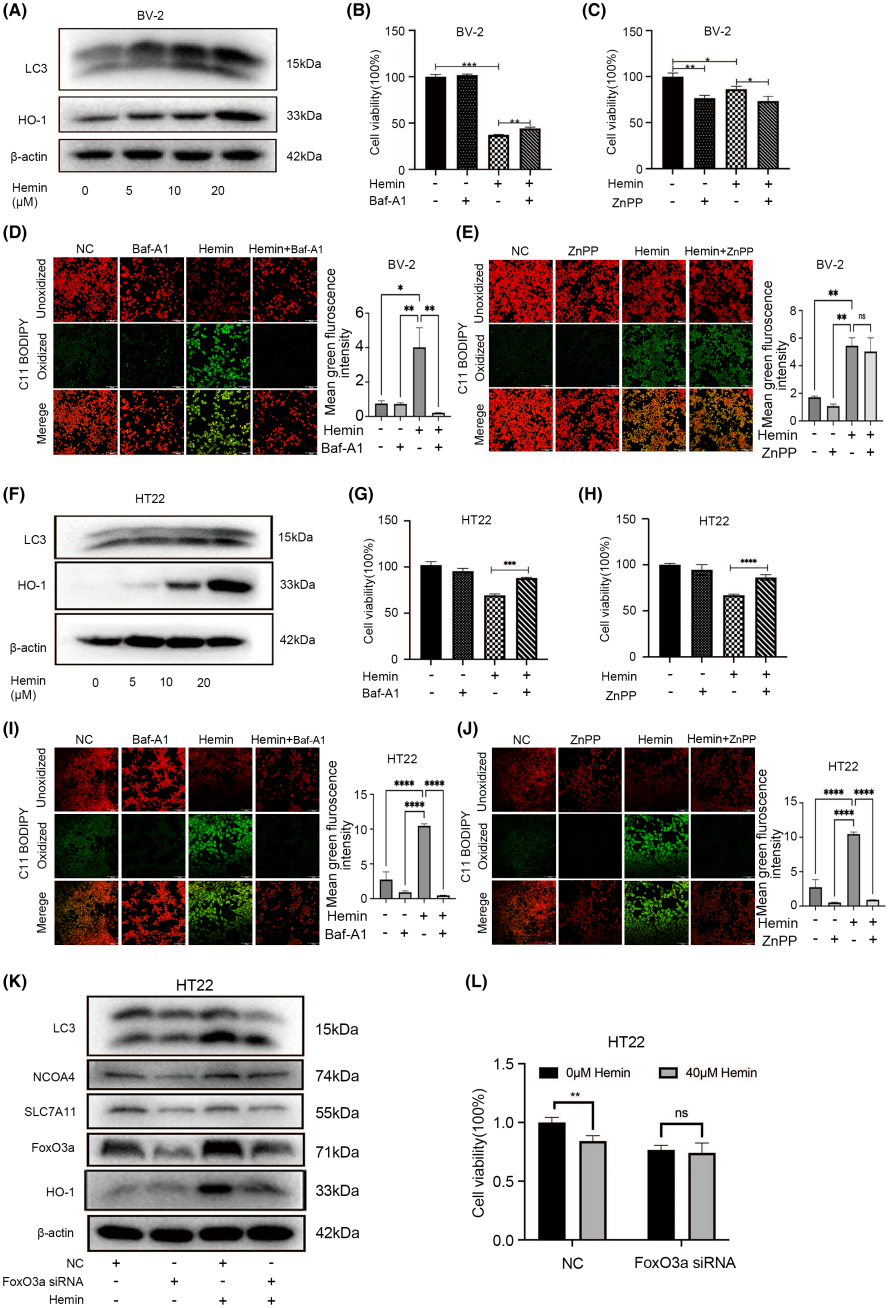
FoxO3a promoted neuronal and microglial ferroptosis induced by hemin through HO‐1 and autophagy pathway. (A) BV‐2 cells were stimulated with different concentrations of hemin. The expressions of LC3‐II/HO‐1 were determined by western blot. (B) Cell viability assay of 10 μM hemin stimulated BV‐2 cells were treated with 5 nM autophagy inhibitor Baf‐A1 and (C) 10 μM HO‐1 inhibitor ZnPP, and cell viability was determined by the CCK‐8 assay. (D‐E) BV‐2 cells were treated as indicated for 24 h, and the accumulation of lipid ROS was assessed by C11‐BODIPY581/591 staining. (F) HT22 cells were stimulated with different concentrations of hemin. The expressions of LC3‐II /HO‐1 were determined by western blot. (G) HT22 cells were treated with or without 20 μM hemin and/or Baf‐A1 (1.25 μM)/(H) HO‐1 inhibitor ZnPP (0.05 μM) for another 24 h. Cell viability was determined by CCK‐8 assay. (I–J) HT22 cells were treated as indicated for 24 h, and the accumulation of lipid ROS was assessed by C11‐BODIPY581/591 staining. (K) HT22 cells were transfected with FoxO3a siRNA for 24 h, then the cells were treated with or without 20 μM hemin for another 24 h. The protein levels of LC3‐II /NCOA4/SLC7A11/FoxO3a/HO‐1 were measured by western blot. (L) After knockdown of FoxO3a in HT22 cells, the cells were stimulated with or without 40 μM hemin, cell viability was determined by CCK‐8 assay. Data were expressed as mean ± SD. *p < 0.05, **p < 0.01, ***p < 0.001.

Autophagy promotes ferroptosis, and FoxO3a is the key regulator of autophagy in multiple cells. In order to explore the underlying mechanism of FoxO3a in hemin‐induced ferroptosis in neuronal and microglial cells, we used different concentrations of hemin to stimulate HT22 cells and BV‐2 cells. Western blot analysis showed that hemin stimulated the expressions of FoxO3a, LC3‐II and HO‐1 in a dose‐dependent manner (Figure 2A,F).

To confirm whether FoxO3a regulates hemin‐induced ferroptosis through induction of HO‐1 and autophagy, HT22 and BV‐2 cells were treated with or without HO‐1 inhibitor (ZnPP) and the autophagy inhibitor (Baf‐A1) after exposure to hemin. Treatment with Baf‐A1 (5 nM) for BV‐2 cells inhibited the ferroptosis induced by hemin (Figure 2B). However, the use of HO‐1 inhibitor ZnPP could not reverse the damage to hemin in BV‐2 cells (Figure 2C). Since the accumulation of lipid peroxidation is a hallmark of ferroptosis, we used C11‐BODIPY581/591 probes to detect lipid peroxidation. As shown in Figure. 2D,E, hemin induced ROS accumulation, whereas treatment of Baf‐A1 but not Znpp reduced lipid peroxidation under hemin exposure. Consistent with the results from CCK‐8 test (Figure2G–H), both Baf‐A1 and ZnPP reversed hemin‐induced ferroptosis of HT22 cells (Figures 2I,J).

To confirm whether FoxO3a played an important role in hemin‐induced ferroptosis, we transfected HT22 cells with specific siRNA to inhibit the expression of FoxO3a. As shown in Figure 2K, FoxO3a siRNA significantly decreased the expression of FoxO3a in HT22 cells. Knockdown of FoxO3a alleviated the enhanced expression of LC3‐II and HO‐1 induced by hemin. The results also showed that knockdown of FoxO3a decreased both basal levels and hemin‐induced ferroptosis‐related protein, such as NCOA4 and SLC7A11, What's more, we found knockdown of FoxO3a in HT22 cells could not cause cell damage after hemin stimulation (Figure 2L). These results suggested that knockdown of FoxO3a might inhibit neuronal and microglial ferroptosis induced by hemin through autophagy and HO‐1 pathway.

### 3.3 FoxO3a modulated the microglial activation phenotype induced by hemin

**FIGURE 3:**
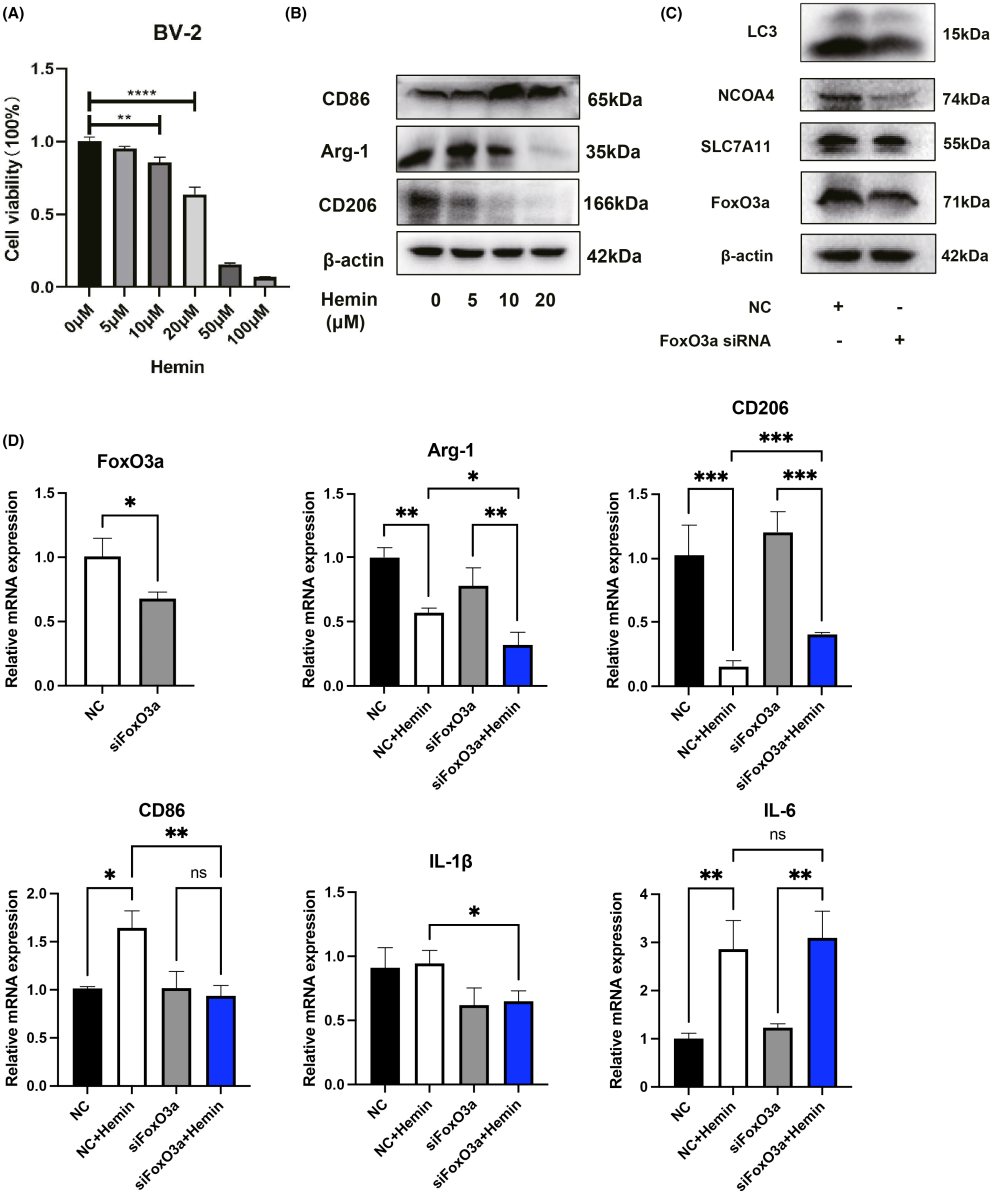
FoxO3a modulated the microglial activation phenotype induced by hemin. (A) BV‐2 cells were treated with different concentrations of hemin. Cell viability was determined by the CCK‐8 assay kit. (B) BV‐2 cells were stimulated with different concentrations of hemin, and the M1/M2 makers of microglial phenotypes were measured by western blot. (C) BV‐2 cells were transfected with FoxO3a siRNA for 36 h, and the expressions of LC3‐II /NCOA4/SLC7A11/FoxO3a were determined by western blot. (D) BV‐2 cells were transfected with FoxO3a siRNA for 24 h, then the cells were treated with hemin for another 24 h. The indicated mRNA levels were determined by qRT‐PCR. Data were expressed as mean ± SD. *p < 0.05, **p < 0.01, ***p < 0.001.

To explore the role of FoxO3a on the hemin‐mediated microglial activation. The polarised M1/M2 markers of microglial cells were determined by western blotting experiments. We found the pro‐inflammatory M1‐type protein CD86 was significantly increased after treatment with different concentration of hemin, while the anti‐inflammatory M2 markers, such as CD206 and Arg‐1, were significantly decreased (Figure 3B).These indicated that hemin promoted the pro‐inflammatory polarisation of microglia to the M1 type. To further study the role of FoxO3a in the regulation of microglia phenotypes (M1 or M2), we transfected BV2 cells with specific siRNAs of FoxO3a. We found FoxO3a siRNA significantly blocked the expression of FoxO3a in BV‐2 cells (Figure 3C). Knockdown of FoxO3a alleviated the enhanced expression of inflammation‐related genes induced by hemin, such as IL‐1β and CD86 (Figure 3D). On the contrary, hemin significantly decreased the levels of anti‐inflammatory genes (CD206 and Arg‐1); the decline mRNA level of CD206 was partially reversed by transfected FoxO3a‐specific siRNA (Figure 3D). These results suggested that knockdown of FoxO3a inhibits microglial M1‐polarisation induced by hemin. In addition, we further used primary microglia cells to confirm these results. We found that knockdown of FoxO3a in microglia cells significantly decreased the expression of the M1‐type markers (CD86 and IL‐1β) in hemin‐stimulated microglia but had no greater effect on IL‐6 expression (Figure 3D). These results suggested that knockdown of FoxO3a inhibits microglial M1‐polarization induced by hemin.

### 3.4 Conditional knockout of FoxO3a in microglia attenuated the neurological deficits and alleviated brain injury induced by ICH

**FIGURE 4:**
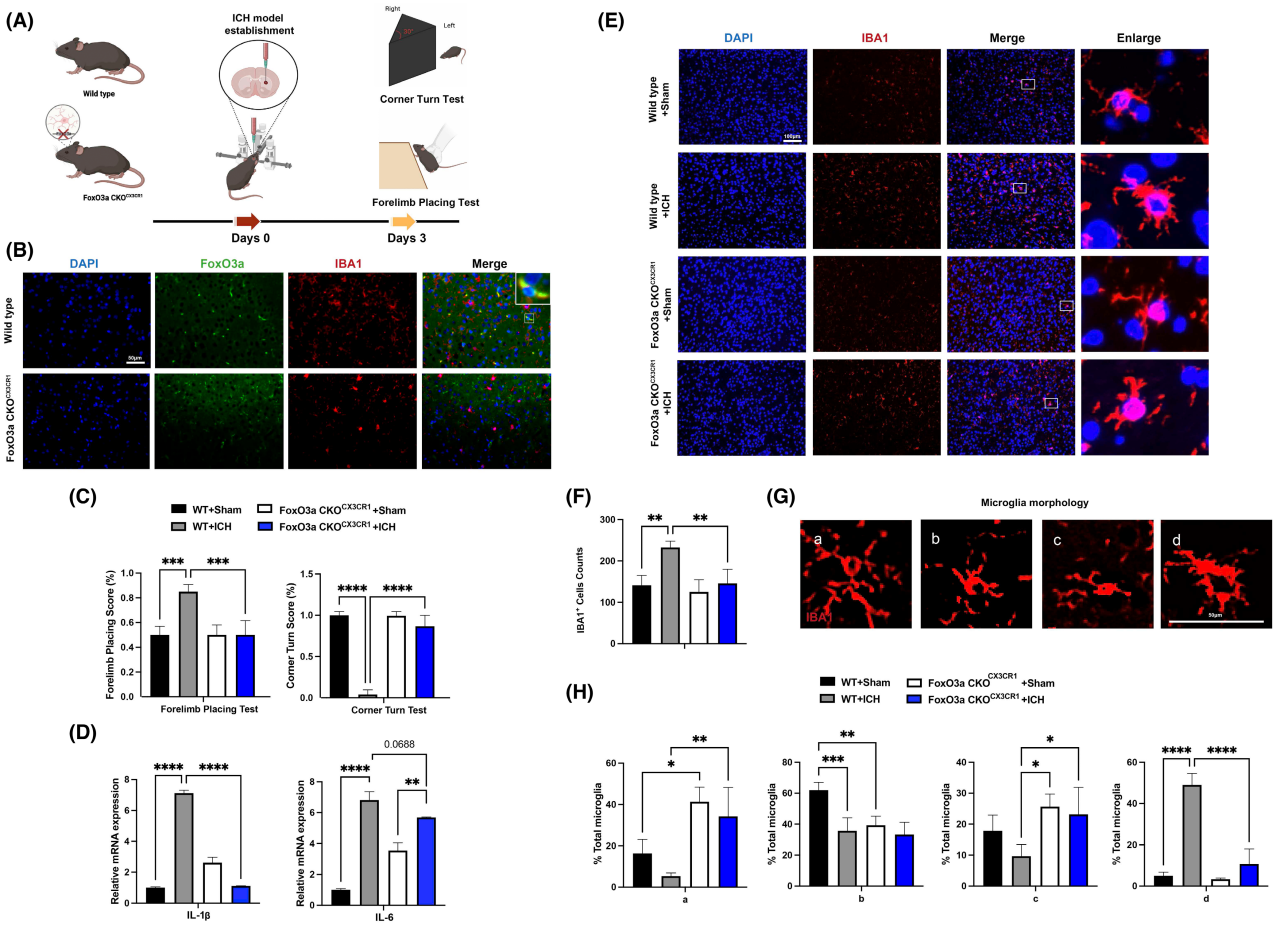
Conditional knockout of FoxO3a in microglia attenuated the neurological deficits and brain injury induced by ICH. (A) Schematic diagram of the experimental design. (B) Representative images showed FoxO3a knockout efficiency in microglia from wild‐type and FoxO3a CKO^CX3CR1^ mice. (C) The forelimb placing test and corner turn test of the FoxO3afl/fl and FoxO3a cKO^CX3CR1^ mice in ICH model by injection of autologous whole blood (*n* = 5). (E) Representative images of IBA1 expression in the striatal area (*n* = 5). (D) The mRNA levels of IL‐1β and IL‐6 were determined by RT‐PCR in the striatum of the FoxO3afl/fl and FoxO3a cKO^CX3CR1^ mice caused by ICH (*n* = 3). (E) Representative pictures of microglia activation phenotype in the striatal region (*n* = 5). (F) The area of the Iba1+ cells in the striatal region. (G) Morphological microglial analysis was represented with the scale for (H), ranking from a (more ramified phenotype) to d (more amoeboid phenotype) was performed (*n* = 5). Data were expressed as mean ± SD. *p < 0.05, **p < 0.01, ***p < 0.001.

To evaluate the functional roles of elevated FoxO3a in microglia after ICH, the experimental design is described in Figure 4A. Firstly, we generated FoxO3a ^fl/fl^:CX3CR1^creER/+^ mice by crossing FoxO3a ^fl/fl^ mice with CX3CR1^creER/+^ mice. In FoxO3a ^fl/fl^ CX3CR1^creER/+^ mice, tamoxifen administration induced the conditional knockout (CKO) of FoxO3a in microglia. As shown in Figure 4B, immunofluorescence staining showed efficient deletion of FoxO3a in microglia of mice. In addition, as shown in Figure 4C, microglial deletion of FoxO3a significantly improved behavioural tests (forelimb placing test and corner turn test).

To confirm the role of FoxO3a in ICH‐induced neuroinflammation, we examined the expression of inflammatory factors in the striatum of mice. We found that mRNA levels of IL‐1β and IL‐6 were significantly increased in the striatum of the ICH‐exposed mice compared with the saline group. FoxO3a CKO ^CX3CR1^ mice prevented an increase of IL‐1β and IL‐6 in the striatum after ICH (Figure 4D). Ionised calcium‐binding adapter molecular 1 (IBA‐1) staining was used for the analysis of microglia activation. We found a prominent increase in the number of activated microglia in the striatum while FoxO3a CKO ^CX3CR1^ mitigated ICH‐induced microglia activation (Figure 4E,F). The microglia underwent morphological changes; the cell bodies enlarged, and more neurites were indicative of M1‐like alternative activation of microglia in the striatum after ICH (Figure 4G). In contrast, microglial deletion of FoxO3a resulted in fewer neurites and smaller cell size in the ICH model (Figure 4G,H).

### 3.5 Conditional knockout of FoxO3a in microglia attenuated the ferroptosis induced by ICH

**FIGURE 5:**
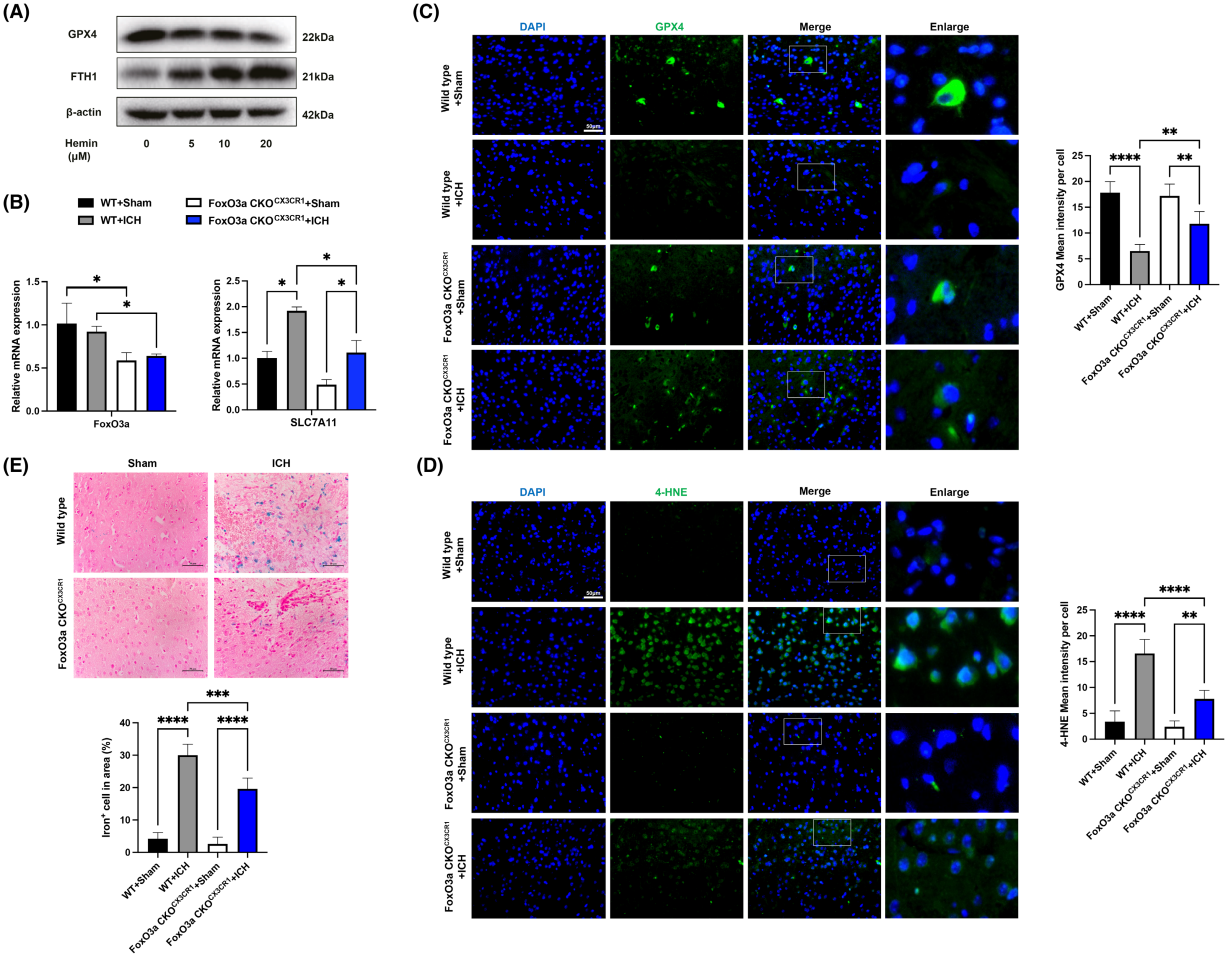
Conditional knockout of FoxO3a in microglia reduces ferroptosis and lipid peroxidation caused by ICH. (A) BV‐2 cells were treated with different concentrations of hemin for 24 h, and then the protein expressions of GPX4 and FHT1 were determined with western blot. (B) The mRNA levels of FoxO3a/SLC7A11 in the striatum of ICH model were determined by qRT‐PCR (*n* = 3). (C) Representative immunofluorescence images of GPX4 expression (*n* = 5). (D) Representative immunofluorescence images of 4‐HNE (*n* = 5) and (E) Representative images of Perl's iron stain in the striatal area of the FoxO3a^fl/fl^ and FoxO3a cKO^CX3CR1^ mice caused by ICH (*n* = 5). Data were expressed as mean ± SD. *p < 0.05, **p < 0.01, ***p < 0.001.

BV‐2 cells were treated with different concentrations of hemin for 24 h. Western blot results showed that the protein expression of GPX4 was decreased while FHT1 was increased in concentration‐dependent manner (Figure 5A). To investigate the effects of conditional knockdown of FoxO3a in microglia on ICH‐induced ferroptosis in vivo. We found the mRNA level of SLC7A11 in the striatum was significantly increased in the ICH model, and this effect was attenuated in microglial FoxO3a‐deficient mice (Figure 5B). Moreover, we further investigated whether loss of FoxO3a in microglia could alleviate the lipid peroxidation caused by ICH mice. Immunofluorescence staining showed that the expression of GPX4 was dramatically decreased in ICH mice while conditional knockout of FoxO3a in microglia reversed this effect (Figure 5C). We stained mouse brain tissues with 4‐HNE and Perl's iron solution. As shown in Figure 5D,E, the expression of 4‐HNE and the accumulation of iron increased in the striatum of mice after ICH, whereas knockout of FoxO3a in microglia significantly reduced the fluorescence expression of 4‐HNE and the accumulation of iron. These results indicated that the role of microglial FoxO3a was involved in ICH‐induced ferroptosis, and deletion of FoxO3a in microglia inhibited ferroptosis.

### 3.6 Knockdown of FoxO3a reduces autophagy and ferroptosis caused by ICH

**FIGURE 6:**
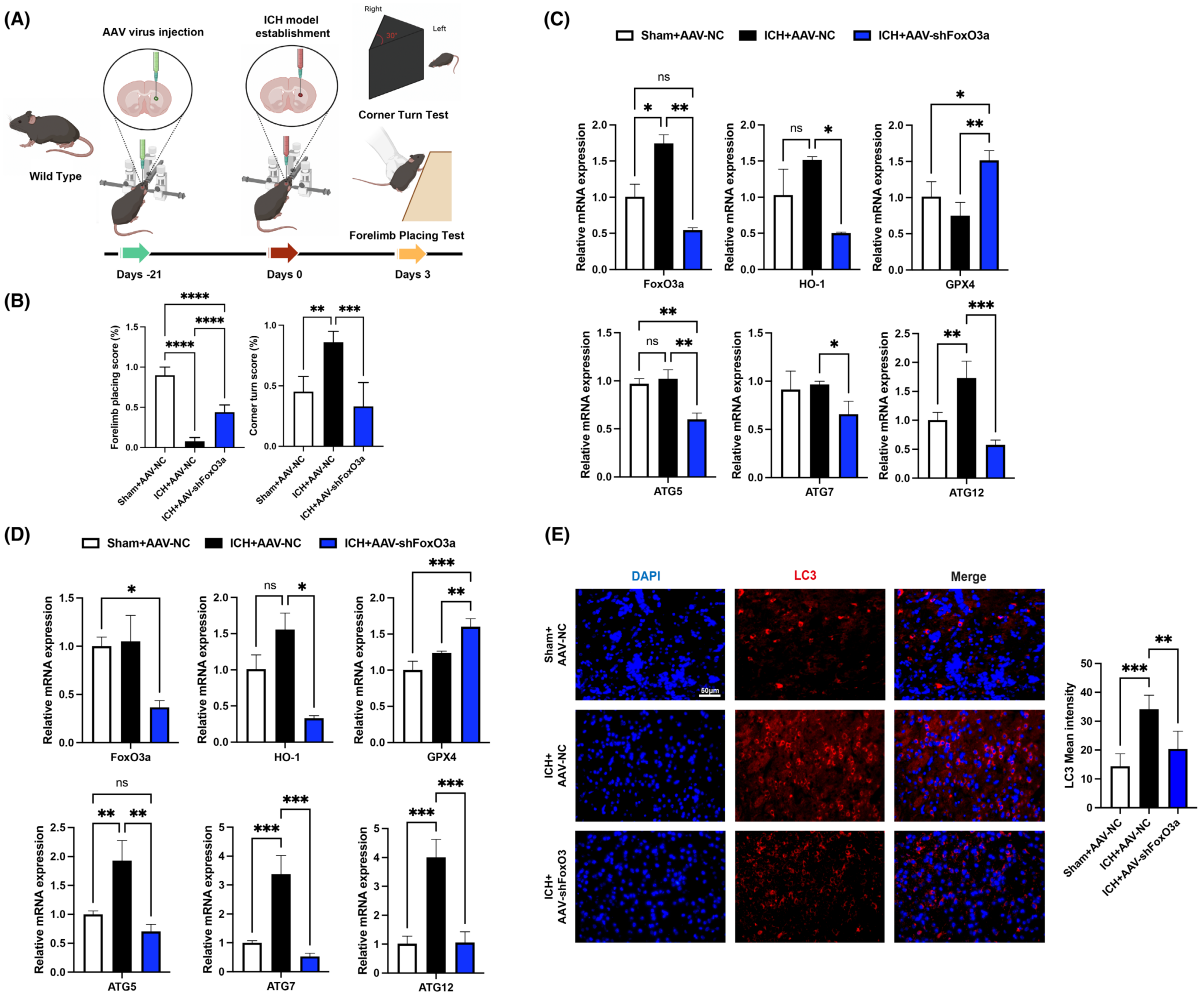
Knockdown of FoxO3a by AAV‐shFoxO3a reduces autophagy and ferroptosis caused by ICH. (A) Schematic diagram of the experimental design. (B) The forelimb placing test and corner turn test of the mice after injection of AAV‐shFoxO3a for 3 weeks in ICH model (*n* = 5). The mRNA levels of FoxO3a, HO‐1, GPX4 and ATG‐related mRNA in the striatum (C) and cortex (D) of mice (*n* = 3). (E) Representative immunofluorescence images of LC3 expression in the striatal area of the mice in ICH model (*n* = 5). Data were expressed as mean ± SD. *p < 0.05, **p < 0.01, ***p < 0.001.

To verify whether knockdown of FoxO3a improved behaviour in mice, forelimb placing tests and corner turn tests were performed after injection of AAV‐FoxO3a shRNA into the striatum of C57BL/6 mice for 21 days in ICH model. We found that mice receiving AAV‐FoxO3a shRNA improved behavioural tests compared with a vehicle control group (Figure 6B). We first confirmed that the FoxO3a mRNA levels were decreased in the striatum and cortex of mice after injection AAV‐FoxO3a shRNA, and in the ICH model, knockdown of FoxO3a by injection of AAV‐FoxO3a shRNA significantly decreased the levels of HO‐1 and autophagy‐related genes (ATG5, ATG7 and ATG12) in the striatum and cortex of mice induced by ICH (Figure 6C,D). Moreover, compared with ICH group, knockdown of FoxO3a significantly increased the expression of GPX4 after ICH (Figure 6C,D).And the immunofluorescence staining results showed the autophagy maker LC3 was significantly decreased in the FoxO3a knockdown group (Figure 6E).

## 4 DISCUSSION

Intracerebral haemorrhage (ICH) is the second largest cause of stroke, second only to ischemic stroke, but it causes a very high rate of cerebrovascular mortality and complications.^1^, ^2^ Although many studies have focused on neurological outcomes after a stroke haemorrhage,^21^, ^22^, ^23^ but precise mechanisms responsible for secondary hippocampal and cognitive loss are not fully elucidated. Here, we established the ICH model by injecting of autologous whole blood into the right basal ganglia of adult mice. The major finding of the present study is that FoxO3a/ATG‐mediated autophagy and that HO‐1 plays an important role in ferroptosis‐induced striatum injury and neuro‐inflammation of ICH.

We made the following observations: (1) FoxO3a participates in the activation of neuro‐inflammation of microglia induced by hemin in vitro and ferroptosis‐induced death of neurons. (2) The activation of microglial FoxO3a may play an important role in the activation of microglia and nerve injury caused by ICH. The specific knockout of microglia FoxO3a significantly inhibits microglial activation and reduces the ferroptosis‐induced striatum injury caused by ICH. (3) FoxO3a participates in the ferroptosis caused by ICH in vitro and in vivo by regulating ATG‐mediated autophagy and the HO‐1 pathway.

Following ICH, the ipsilateral hippocampal damage together with a subsequent decline of cognitive function.^19^ HO‐1‐positive cells around the hematoma was associated mostly with microglia. To a lesser extent, the neuron loss during ageing and age‐related neurodegenerative diseases may be related to iron deposition in neurotoxicity.^6^, ^10^, ^24^ In the specific environment of ICH, the expression pattern of HO‐1 changes after hemorrhagic injury.^10^, ^25^ After cerebral haemorrhage, HO‐1 is mainly concentrated in microglia and a few neurons.^24^ The overexpression of HO‐1 may play its biological role by changing the function of microglia. In this study, we found endogenous expression of HO‐1 were increased following ICH. Moreover, FoxO3a‐deficient microglia in mice significantly increased the expression of GPX4, while the mRNA levels of autophagy‐related genes and HO‐1 expression were decreased. We speculate that FoxO3a and HO‐1 play an important role in microglial activation and the process of ICH.

Intracerebral haemorrhage shares features of ferroptosis and necroptosis; hemin is an oxidized product of haemoglobin from lysed red blood cells, leading to secondary injury.^26^, ^27^ To underlying the molecular mechanisms attributable to secondary injury by hemin. Here, we established the ICH model by hemin‐induced neuronal cells in vitro and injection of autologous whole blood into the right striatum of mice in vivo. We found that the endogenous expression of FoxO3a and HO‐1 was increased after ICH. We also found that FoxO3a was highly co‐located with neurons and microglia but not astrocytes after ICH. Conditional knockout of FoxO3a in microglia for mice attenuated neurological deficits and microglial activation; at the same time, it also inhibited the ferroptosis in the area around the hematoma after ICH. Previous studies showed that knocking down FoxO3a in macrophages inhibited autophagy and inhibited the increase of inflammatory cytokines in macrophages induced by LPS.^28^ In this study, FoxO3a‐deficient microglia in mice significantly increased the expression of GPX4 while reducing the mRNA levels of HO‐1, IL‐6 and IL‐1β. Recently, autophagic flux and expression of autophagy‐related genes were significantly suppressed by LPS through the PI3K/Akt/FoxO3a signalling pathway in microglia.^29^ We demonstrate that microglial FoxO3a/ATG‐mediated autophagy and HO‐1 play an important role in neuro‐inflammation and the process of ICH. These results suggest that this will provide new ideas for the treatment of ICH. On the other hand, Sirt3 overexpression activated FoxO3a and then induced an increase in the expression of anti‐oxidants (Cat and MnSOD) in microglia,^30^ suggesting that Sirt3 regulates ROS by inducing the expression of antioxidants via activation of FoxO3a. The above studies suggested that the activity of FoxO3a for anti‐oxidant and anti‐inflammatory effects in microglia might be bidirectional and play different roles under different physiological conditions.

Previous studies showed that autophagy promotes ferroptosis by degradation of ferritin, knockout or knockdown of Atg5 (autophagy‐related 5) and Atg7 inhibited erastin‐induced LC3‐II and limited erastin‐induced ferroptosis.^31^ Inhibition of PTEN ameliorates secondary hippocampal injury and cognitive deficits through AKT/FoxO3a/ATG‐mediated autophagy after ICH.^18^, ^19^ ICH commonly causes secondary hippocampal and striatum damage. However, whether FoxO3a/ATG‐mediated autophagy is involved in ferroptosis‐induced striatum injury of ICH remains largely unknown. In this study, we found knock‐down of FoxO3a alleviated the enhanced expression of LC3‐II and HO‐1 induced by hemin. Knockdown of FoxO3a significantly downregulated the expression of ATG5/ATG7/ATG12 /HO‐1while upregulated the expression of GPX4 in the striatum after ICH. These results reveal that knockdown of FoxO3a might inhibit neuronal and microglial ferroptosis‐induced by hemin through HO‐1 and the autophagy pathway. The immunofluorescence staining results showed LC3 was also decreased in the FoxO3a knockdown group. The results also showed that knockdown of FoxO3a decreased both basal levels and hemin‐induced ferroptosis‐related protein, such as NCOA4 and SLC7A11. These results consistent with previous studies that ATG5‐ATG7‐NCOA4 autophagic pathway promotes ferroptosis by degradation of ferritin.^31^ Many studies provide direct evidence supporting the hypothesis that inhibition of SLC7A11 induces ferroptosis and aggravates ischemia.^32^, ^33^ Interestingly, we found the mRNA level SLC7A11 in the striatum were significantly increased in the ICH model, and this effect was reversed in microglial FoxO3a‐deficient mice. High‐expression SLC7A11‐cells were more resistant to ferroptosis than low‐expression SLC7A11‐cells, we found an inverse correlation between SLC7A11 expression and ferroptosis sensitivity in mice model of ICH. The reasons may be attributed to SLC7A11 promotes long‐lasting glutamate excitotoxicity.^34^ All these results identified FoxO3a as a key activator of autophagy in mice model of ICH, and FoxO3a‐mediated autophagy and HO‐1 was essential for the ferroptosis induced‐brain injury in response to ICH.

## 5 CONCLUSION

We demonstrate, for the first time, that hemin promoted ferroptosis of neuronal cells via FoxO3a/ATG‐Mediated Autophagy and HO‐1 signalling pathway. FoxO3a/ATG‐mediated autophagy and HO‐1 plays an important role in microglial activation and process of ICH, identifying a new therapeutic avenue for the treatment of ICH.

## AUTHOR CONTRIBUTIONS

**Rikang Wang:** Supervision (equal); writing – original draft (equal); writing – review and editing (equal). **Zhi Liang:** Investigation (equal); methodology (equal). **Xiaoyan Xue:** Investigation (equal). **Hua Mei:** Formal analysis (equal); investigation (equal). **Lianru Ji:** Investigation (equal). **Wenjin Chen:** Investigation (equal); methodology (equal). **Bocheng Wang:** Investigation (equal); methodology (equal). **Chao Gao:** Investigation (equal); methodology (equal). **Shun Yuan:** Investigation (equal); project administration (equal). **Tao Wu:** Writing – review and editing (equal). **Hui Qi:** Validation (equal). **Suifa Hu:** Formal analysis (equal). **Yi Li:** Supervision (equal). **Yonggui Song:** Supervision (equal). **Rifang Liao:** Funding acquisition (equal); supervision (equal). **Baodong Chen:** Supervision (equal).

## FUNDING INFORMATION

This work is supported by the National Natural Science Foundation of China (81803536; 82171351), Shenzhen Basic Research Projects (JCYJ20190822090801701) and Scientific Research Foundation of Peking University Shen Hospital (KYQD202100X; KYQD2023254).

## CONFLICT OF INTEREST STATEMENT

The authors declare no conflicts of interest.

## DATA AVAILABILTY STATEMENT

All data generated or analysed during this study are included in this published article.

## ETHICS STATEMENT

The animal experiments were performed according to internationally followed ethical standards and approved by the research ethics committee of Jiangxi University of Chinese Medicine.
